# Magnetoelectric Nanoparticle‐Based Wireless Brain–Computer Interface: Underlying Physics and Projected Technology Pathway

**DOI:** 10.1002/advs.202524329

**Published:** 2026-03-28

**Authors:** Elric Zhang, Max Shotbolt, Mostafa Abdel‐Mottaleb, Shawnus Chen, Victoria Andre, Jieyuan Tian, Jonathan Shulgach, Max Murphy, Brian Noga, Ping Liang, Darcy Griffin, Douglas Weber, Marta Pardo, Salvador Pane, Sakhrat Khizroev

**Affiliations:** ^1^ College of Engineering University of Miami Coral Gables Florida USA; ^2^ Institute of Robotics and Intelligent Systems Federal Institute of Technology at Zurich (ETHZ) Switzerland; ^3^ College of Engineering and Neuroscience Institute Carnegie Mellon University Pittsburgh Pennsylvania USA; ^4^ The Miami Project to Cure Paralysis and Department of Biochemistry and Molecular Biology Miller School of Medicine University of Miami Miami Florida USA; ^5^ Cellular Nanomed Irvine California USA; ^6^ The University of Valencia Valencia Spain

**Keywords:** brain–computer interface, magnetoelectric nanoparticles, neuromodulation, neural recording, neurotechnology

## Abstract

Magnetoelectric nanoparticles (MENPs) provide a fully wireless and minutely invasive platform for bidirectional brain–computer interfaces (BCIs) by locally transducing magnetic fields into electric fields, and vice versa. The achievable spatial and temporal resolutions are governed by the control of magnetic field energy at the nanoparticle level. Since the introduction of the MENP concept a decade and a half ago, independent studies have demonstrated MENP‐mediated neural activation in vitro and in vivo, establishing a strong proof of concept for wireless neuromodulation. In contrast, MENP‐based neural recording remains largely theoretical, with existing models indicating that in vivo implementation is feasible. However, progress toward scalable and reliable MENP‐based BCIs is hindered by an incomplete understanding of the nonlinear physics governing MENP operation and nanoparticle–cell interactions. This study addresses this gap by developing a comprehensive theoretical framework that explicitly incorporates nonlinear effects and correlates neuromodulation predictions with available experimental data. The analysis identifies nanoparticle properties and magnetic field amplitude and frequency as key performance determinants. Properly engineered MENPs are predicted to enable deepbrain and cortical neuromodulation and recording with submillimeter spatial resolution and millisecondscale temporal precision, offering a pathway toward clinically viable BCIs without implanted electrodes or genetic modification.

## Graphical abstract

Magnetoelectric nanoparticles (MENPs) enable fully wireless, minutely invasive neuromodulation, and potentially neural recording, by converting magnetic into electric and, conversely, electric into magnetic fields, respectively, at high spatiotemporal resolution. This work develops a nonlinear theoretical framework, validated against experiments to date, identifying key material and field parameters to optimize MENP‐based BCIs and advance them toward clinically scalable brain interfaces.

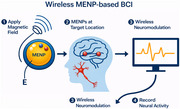

## 1 Introduction

Brain–computer interfaces (BCIs) capable of highresolution, bidirectional neural communication would be transformative for both neuroscience research and the clinical treatment of neurological diseases [1]. High‑resolution BCIs are essential for developing therapies for currently untreatable neurological diseases and for elucidating fundamental mechanisms underlying the operation of biological neural circuits. Traditional wired neurotechnology solutions have produced numerous clinically viable treatments for Parkinson's, Epilepsy, and other neurodegenerative diseases and even brain tumors [2, 3, 4]. However, deep understanding of the underlying mechanisms of these treatments remains opaque. Moreover, electrode‐based approaches require invasive surgical implantation, pose risks of infection, and produce off‐target effects due to electrical current spreading beyond the intended neural populations. These limitations restrict their use to patients with severe, drug‐refractory conditions [5].

Over the past two decades, there have been rapidly growing efforts to develop less invasive neuromodulation alternatives, including optogenetics, focused ultrasound, magnetic stimulation, and other particle‐based approaches [5, 6, 7, 8, 9]. Among these, magnetoelectric nanoparticles (MENPs) have recently attracted significant attention as promising agents for wireless neural interfacing [10, 11, 12, 13, 14, 15, 16, 17, 18]. This is due to their unique ability to replicate the effects of electrode‐based stimulation by converting remote magnetic fields into localized electric fields, and conversely, translating local electric fields into detectable magnetic signals.

MENPs are a class of nanomaterials typically consisting of a core–shell structure combining magnetostrictive and piezoelectric materials [19]. When exposed to a magnetic field, the magnetostrictive core strains the piezoelectric shell at the core–shell interface, generating a highly localized electric field capable of modulating membrane potentials. Currently, MENPs are capable of sub‐millimeter spatial resolution and in the future, potentially, capable of a single‐neuron precision. Additionally, the reverse process (conversion of local electric fields into magnetic signals) enables remote detection of neural activity via external magnetometers, providing a pathway to fully wireless, bidirectional BCIs. This bidirectional capability distinguishes MENPs from other wireless approaches. Unlike optogenetics, MENPs require no genetic modification of the target tissue and do not necessitate surgical procedures to deliver light into the brain. Unlike transcranial magnetic stimulation, they generate electric fields at the nanoscale rather than centimeter‐scale, offering the potential for single‐neuron resolution.

Since first introducing this concept over a decade ago [10], our group and others have demonstrated MENPmediated neural activation in vitro as well as deepbrain stimulation in rodent models. Collectively, these studies establish a strong proof of concept for fully wireless neuromodulation using MENPs. Nevertheless, important gaps remain in the understanding, refining, and optimization of MENP‐based neural interfaces, limiting reliable and scalable applications.

The magnetoelectric response of core–shell nanoparticles is inherently nonlinear, arising from the complex interplay among the nanoparticles’ properties, including magnetic hysteresis, superparamagnetic transition and ferroelectric polarization dynamics, and the nanoparticle‐cell interaction [19]. How these nonlinearities influence neuromodulation and neural recording efficacy, and whether they can be harnessed for selective control of neural activity, has not yet been systematically investigated.

Focusing on the more mature neuromodulation mode, this paper addresses these gaps. First, we present a theoretical perspective accounting for the nonlinear magnetization–strain–polarization coupling at the core–shell interface and derive expressions that relate MENP properties to neuromodulation probability and recording signaltonoise ratio. Next, we show that targeted improvements in magnetoelectric coefficient and membrane affinity significantly enhance neuromodulation efficacy, validating the framework's value for guiding materials optimization. Finally, we outline a physics‐based pathway for translating MENPenabled wireless BCIs toward clinical application.

## 2 Theory of Non‐Linear Characteristics

**FIGURE 1:**
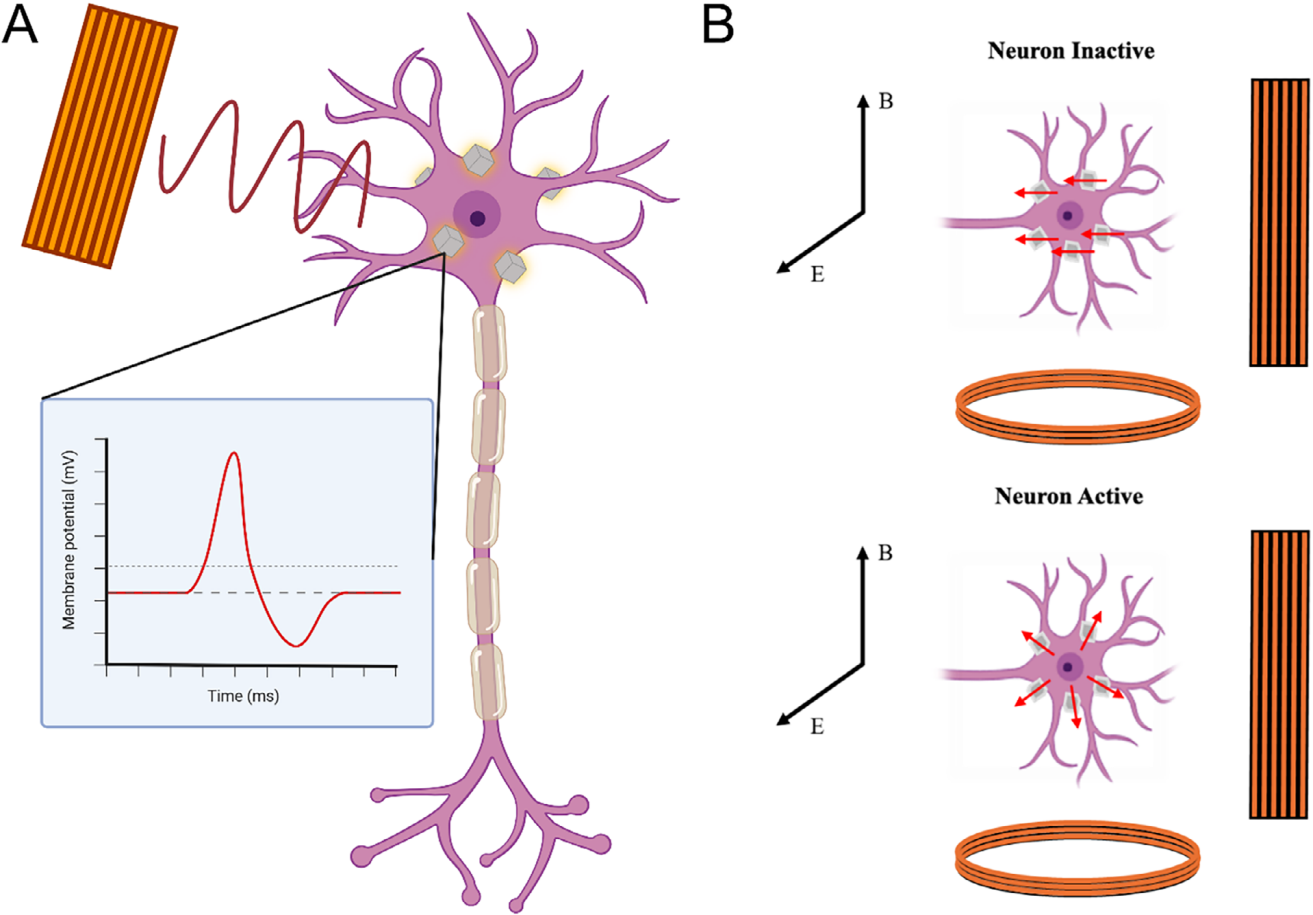
Physics of neuromodulation and recording. (A) In the neuromodulation mode, due to the direct ME effect of MENPs anchored to the membrane, an applied magnetic field induces a local electric field, which in turn controls ion channels and thus neuromodulation. (B) In the active recording mode, when a neuron is at rest, an applied small AC magnetic field keeps the magnetization of the nanoparticles under measurement synchronized (top). If the neuron fires, due to the converse ME effect, a local electric field generated at the membrane breaks the magnetization synchronization. This transient magnetization change is detected via a magnetic field sensor.

The MENP‐BCI is driven by the ME effect of MENPs. This effect allows for the conversion of magnetic fields into local electric field and, vice versa. In turn, these two field conversions, known as the direct and converse ME effects, can provide wireless neuromodulation and recording modes, respectively (Figures 1A,B), thus enabling bidirectional wireless BCI.

The ME effect in multiferroic systems is conventionally described by linear constitutive relations derived from Landau–Ginzburg–Devonshire (LGD) theory. Expanding the free energy as a power series in electric and magnetic fields yields [20]:

(1) $$\begin{array}{c} \;\Delta P_{i}=\alpha_{i}\;H_{i}\; \end{array}$$

(2) $$\begin{array}{c} \;\Delta M_{i}=\alpha_{i}\;E_{i}. \end{array}$$

where *P_i_* and *M_i_* are the *i‐*th coordinate components (*i* = *x,y,z*) of the MENP's polarization and magnetization, respectively, *E_i_* and *H_i_* are the *i*‐th components of the electric and magnetic fields, respectively, and α_*i*_ ≡ α_*ii*_ is *i*‐th diagonal component of the magnetoelectric coupling tensor (assuming a coordinate system aligned with the principal axes). While Equations (1) and (2) provide useful insight into field interconversion, they fail to capture the physics of core–shell MENPs for two reasons. First, these nanoparticles are not single‐phase multiferroics; instead, their ME effect arises from strain‐mediated coupling between distinct magnetostrictive and piezoelectric components. Second, both components, due to the magnetic hysteresis in the core and ferroelectric hysteresis in the shell, exhibit highly nonlinear responses that fundamentally define neuromodulation and recording efficacy, respectively. This section aims to describe a framework accounting for these nonlinearities.

### 2.1 MENP‐BCI: Neuromodulation Mode

#### 2.1.1 The Magnetoelectric Cascade in Core–Shell Nanoparticles

When an external magnetic field is applied to a MENP, the direct ME effect induces electric polarization, generating a local electric field in the nanoparticle's vicinity (Figure 1A) [21]. Unlike single‐phase multiferroics, core–shell MENPs generate the ME effect through strain‐mediated coupling between mechanically distinct phases. The effective ME coefficient is the product of the magnetostrictive coefficient of the core and the piezoelectric coefficient of the shell. Hence, the nonlinearities in either phase propagate directly to the overall response.

**FIGURE 2:**
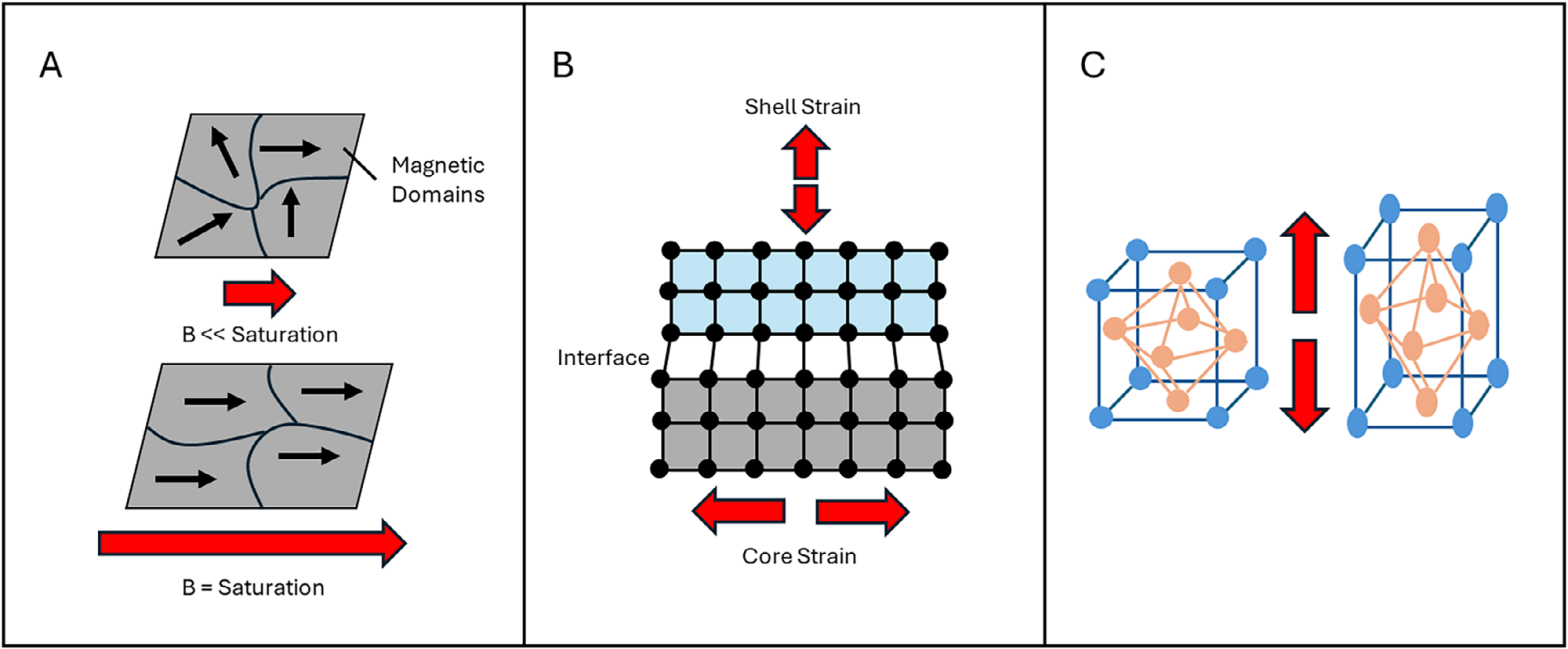
Sequence of physical processes in the core–shell MENP structure during the neuromodulation event: (A) an externally applied magnetic field changes the magnetization of the core according to its M–H hysteresis loop; (B) the magnetization change causes a strain which propagates through the lattice‐matched interface to the shell; (C) the transferred strain induces a dipole electric moment, thus generating a local electric field.

During each stimulation event, four steps occur in sequence (Figure 2). First, the applied magnetic field alters the magnetization of the magnetostrictive core according to its M–H hysteresis loop. Second, the magnetization change induces strain in the core's crystallographic structure via the magnetostrictive effect. Third, because the core and shell share a lattice‐matched interface, strain propagates from the core into the piezoelectric shell. Finally, the piezoelectric effect converts the transferred strain into an electric dipole moment, generating a local electric field.

This cascade reveals why maximizing stimulation efficacy requires optimizing each step. The magnetic field must produce large magnetization changes (Step 1), the core must efficiently convert magnetization into strain (Step 2), the interface must transfer strain without loss (Step 3), and the shell must exhibit strong piezoelectric response (Step 4). The nonlinear characteristics of magnetic and ferroelectric hysteresis are particularly critical and are analyzed in detail below.

#### 2.1.2 The Role of Debye Screening

For the ME effect to modulate neural activity, the MENP must be anchored within the Debye screening length of the neuronal membrane. In electrolyte solutions such as the intracellular and extracellular fluids, free ions redistribute in response to local electric fields, screening charges over a characteristic distance known as the Debye length. Under physiological conditions (ionic strength ∼150 mM), the Debye length is approximately 0.7–1 nm [22]. Electric fields generated by a dipole source decay to negligible levels beyond this distance due to ionic screening, with a millisecond time interval.

**FIGURE 3:**
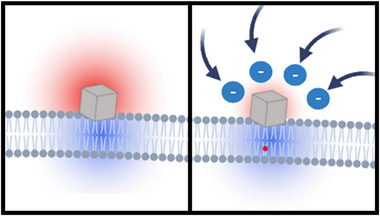
Illustration of the physics of generating a strong electric field across the membrane when a MENP is in the immediate vicinity of the membrane. (Left) Because a MENP is a dipole, prior to screening by free moving ions due to the Debye effect, it has positive and negative charges on its top and bottom sides, respectively. (Right) After screening, due to the Debye effect, the top side's electric field contribution is effectively cancelled out by the free charges, resulting in a strong electric field across the membrane by the uncompensated charge of the bottom side.

Consider a MENP in contact with the neuronal membrane, with its polarization axis‐oriented perpendicular to the membrane surface (Figure 3). The ME‐induced polarization creates positive and negative surface charges on opposite faces of the nanoparticle. The outward‐facing charge (distal to the membrane) is rapidly screened by free ions in the surrounding medium, contributing negligibly to the transmembrane field. However, the inward‐facing charge (proximal to the membrane) lies within the Debye length of the lipid bilayer and cannot be fully screened. This fully exposed (non‐screened) charge generates a field *E_z_* across the membrane:

(3) $$E_{z}=P_{z}/2=\alpha_{z}H_{z}/2$$

where *z* is the orientation perpendicular to the membrane surface. The factor of 1/2 arises because only the membrane‐proximal face contributes to the transmembrane field, while the distal face is screened (Figure 3).

To evaluate the magnitude of this effect, consider typical parameter values, e.g., α_*z*_ = 10 V/Oe/cm and H_z_ = 1 kOe. Equation (3) yields a field from one nanoparticle on the order of 5000 V/cm [19]. For comparison, the resting transmembrane field in neurons is approximately 70 mV across a 5‐nm membrane, corresponding to ∼140,000 V/cm. Thus, MENP‐generated fields can represent a significant perturbation to the local membrane potential–‐sufficient to gate voltage‐sensitive ion channels if delivered to enough membrane area by multiple synchronized nanoparticles.

#### 2.1.3 Effect of Superparamagnetic Transition on Neuromodulation

**FIGURE 4:**
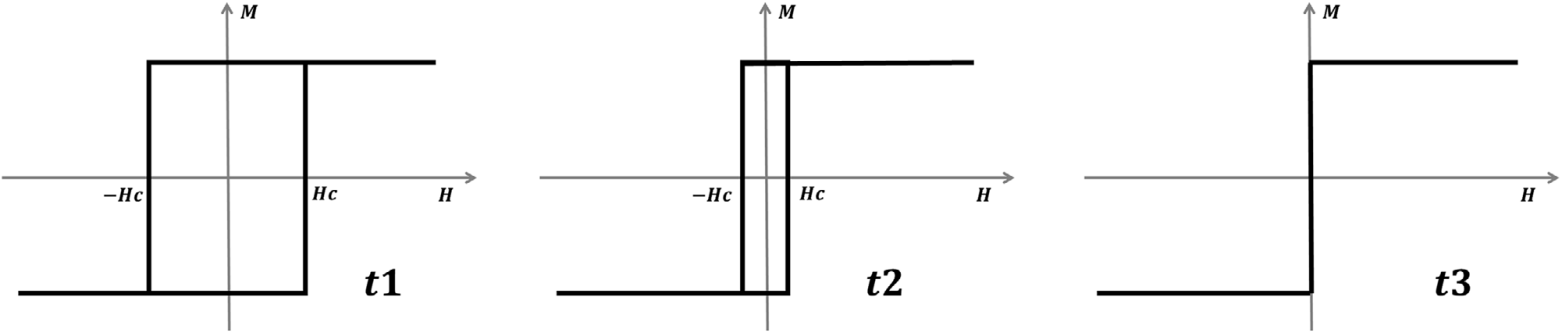
Superparamagnetic transition depending on the measurement time constant. M–H hysteresis loops are measured with three different time constants, *t*1<*t*2<t3, so that *t*1<*t*2< ***τ*** <t3, where ***τ*** is the superparamagnetic transition constant. Among the three loops, only the one measured with the constant t3 indicates the superparamagnetic state. The coercivity field, *H*_C_, depends on the measurement constant.

Maximizing the local electric field generated by MENPs requires maximizing the change in core magnetization during each stimulation cycle. Because the magnetization–field relationship follows an M–H hysteresis loop, the achievable magnetization swing depends not only on the applied field amplitude but also on the nanoparticle's magnetic anisotropy and the effective measurement time set by the stimulus frequency. In core–shell MENPs, this dependence is critical, since the ME coefficient effectively scales with the magnetostrictive response of the core. To simplify the analysis, the average MENP can be modeled as a single‐domain particle with uniaxial anisotropy, described by the Stoner–Wohlfarth model [23]. In this framework, the anisotropy field, 𝐻_𝐾,_ is an intrinsic field determined by the material's magnetocrystalline anisotropy that defines the field required to rotate the magnetization away from its easy axis. In contrast, the coercive field, 𝐻_𝐶,_ is an extrinsic quantity that characterizes the field needed to reverse the magnetization and set the width of the full hysteresis loop. 𝐻_𝐶_ is always a fraction of 𝐻_𝐾_ and depends on the angle between the applied field and the easy axis and on the effective measurement time (or equivalently, stimulus frequency). In the quasi‐static limit (very slow measurement, near‐DC frequency), due to thermal fluctuations, 𝐻_𝐶_ is driven to zero. Whereas in the high‐frequency or fast‐measurement limit, 𝐻_𝐶_ approaches its maximum value along the selected orientation, on the order of 𝐻_𝐾._ The time (or frequency) dependence of coercivity is governed by the superparamagnetic transition. The relevant quantity is the stability ratio SR = $\frac{KV}{k_{B}T}$, where, *K* and *V* are the core's magnetic anisotropy and volume, respectively, *k_B_* is the Boltzmann constant and *T* is the ambient temperature [24]. SR compares the anisotropy energy barrier to thermal energy and determines the Néel relaxation time τ of the nanoparticle's magnetization, which scales approximately as

(4) $$\tau \propto \tau_{0}e^{SR}$$

where τ_0_ is the material‐dependent attempt time, on the order of 1 ns [24]. For a given stimulus frequency 𝑓, the effective measurement time is set by roughly half the AC period, 𝑡_meas_∼1/(2𝑓) When 𝑡_meas_≪𝜏, the magnetization is effectively “frozen” on the measurement timescale, producing a finite coercive field and a well‐defined hysteresis loop. When 𝑡_meas_≫𝜏, the nanoparticle enters the superparamagnetic regime where the magnetization thermally fluctuates and closely follows the applied field, causing the hysteresis loop to collapse and 𝐻_𝐶_ to vanish (Figure 4).

This exponential dependence on 𝑆𝑅 implies that small changes in core size or anisotropy can dramatically alter τ and, therefore, the operating regime. For example, in a cobalt ferrite core (anisotropy on the order of 10^6^ J/m^3^) with a characteristic size of ∼10 nm, reducing the core size by a few nanometers can shift the Néel relaxation time from years to seconds or milliseconds. At typical stimulation frequencies near 60 Hz (measurement time ∼10 ms), a 7 nm core may still exhibit a finite hysteresis loop, while a 6 nm core can already be superparamagnetic on this timescale. In the latter case, the magnetostrictive response, and thus the magnetoelectric effect, collapses, since magnetostriction requires a well‐defined coupling between spin and lattice that is effectively lost when the magnetization freely fluctuates. These considerations show that both nanoparticle dimensions and stimulation frequency must be co‐designed to keep MENPs in a regime with finite coercivity and strong magnetostrictive response. However, there is a trade‐off. On one hand, it is desirable to reduce the required field strength, particularly for the purpose of creating wearable devices, in turn leading to the need to reduce the coercivity field. On the other hand, operating too close to the superparamagnetic limit reduces the effective full M–H loop area and, therefore, the strain and electric field generated per cycle. It can be noted that applying an AC field with a strength smaller than H_C_ would lead to evoking only a minor M‐H loop, according to the Stoner–Wohlfarth model [23]. Following the minor loop would result in the maximum magnetization change being significantly smaller than the saturation magnetization, in turn leading to a significantly reduced induced electric field, thus lowering the neuromodulation efficacy [25]. Hence, properly choosing core size and anisotropy so that τ matches or slightly exceeds the stimulation timescale ensures robust hysteresis and maximizes the local electric field delivered to the membrane, while minimizing the required magnetic field strength. Importantly, this tradeoff can be optimized by adjusting the MENP's material composition and geometry to meet the requirements of each specific application.

#### 2.1.4 Prior Published Experiments Demonstrating the Control of the Magnetoelectric Effect Via the Superparamagnetic Transition

The first proof‐of‐concept experiment to use the above‐described concept of the superparamagnetic transition for controlling the magnetoelectric effect of MENPs was described in a 2020 study [19]. In this study, two different compositions of core–shell MENPs in the same size range, 20–30 nm, were equivalently compared by measuring the ME effect at the single‐nanoparticle level via a direct nanoprobe method. The two compositions, CoFe_2_O_4_@BaTiO_3_ and NiFe_2_O_4_@BaTiO_3_, respectively, had different magnetostrictive cores, while having the same piezoelectric shell. The two magnetostrictive cores had the magnetocrystalline anisotropy on the order of 10^6^ and 10^5^ J/m^3^, respectively, leading to significantly different values of the above superparamagnetic transition constant τ, according to the above theory. As a result, unlike the cobalt‐based MENPs, the Ni‐based MENPs in this size range were in the superparamagnetic state in the DC case, thus not displaying any ME effect. Only, when the frequency of the applied magnetic field exceeded approximately 100 Hz, the magnetoelectric effect of the Ni‐based MENPs became comparable to that of the cobalt‐based MENPs, in agreement with the above theory. The experiment proved that MENPs of specific compositions and sizes could be used to provide neuromodulation and recording, wirelessly controlled at desired frequencies. Another major advantage of the Ni‐based MENPs was the significantly lower field, by a factor of ten, required for neuromodulation.

#### 2.1.5 How MENPs’ Physical Properties Can be Used to Control Neuromodulation

To relate the MENP physics to functional neuromodulation, the key quantity of interest is the probability that a given magnetic pulse will trigger a neuronal firing event. Assuming MENPs are in direct contact with the membrane and act as integral components of the local ion‐gating environment, this probability can be approximated by the probability that a sufficient number of nanoparticles undergo a full magnetization reversal during a stimulation cycle.

**FIGURE 5:**
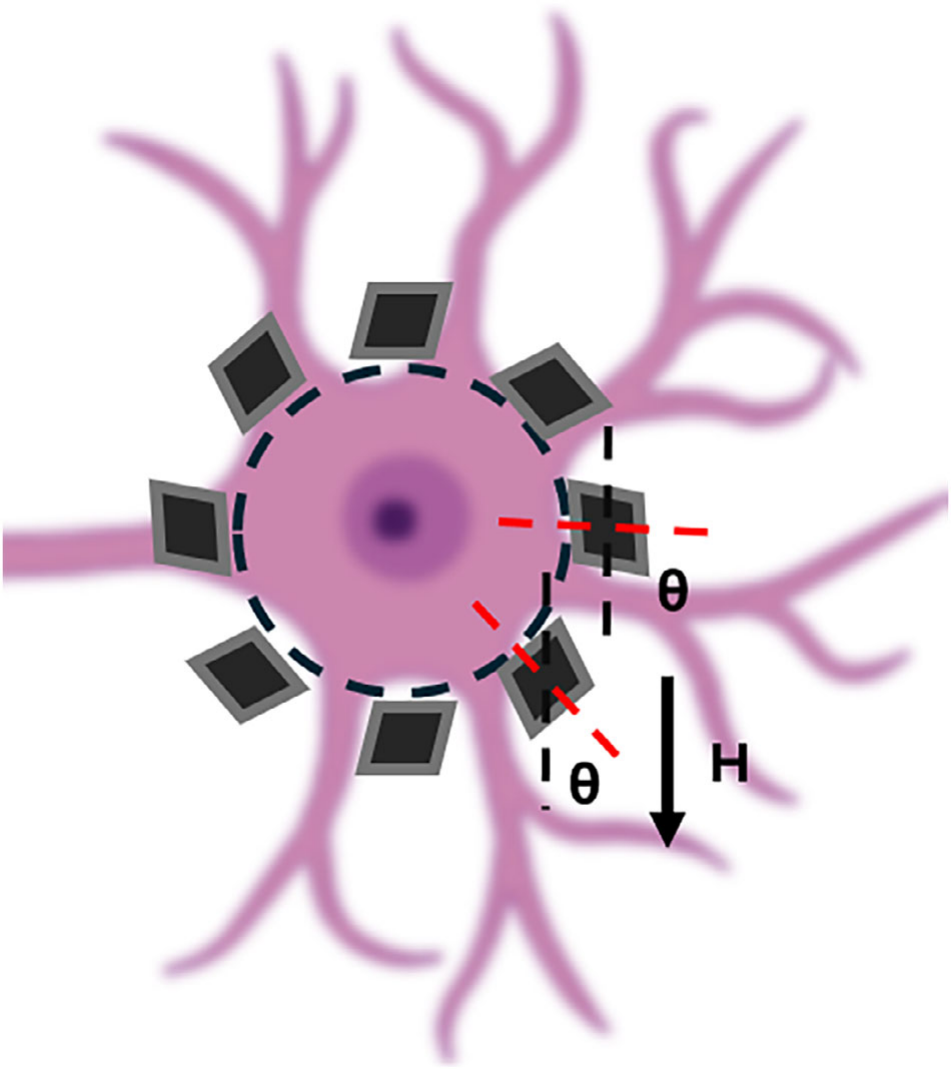
Spherical model of an axon with MENPs uniformly distributed over the membrane surface. The angle (*θ*) between the applied field and the easy axis (e.a.) of a MENP, defined by the magnetic anisotropy of its core, varies from 0 to 360 degrees over the surface. This angle affects the coercivity field of the nanoparticle.

Under the single‐domain, uniaxial anisotropy approximation, the probability that an individual nanoparticle's magnetization fully reverses during a pulse of amplitude 𝐻 can be written as

(5) $$P_{M}∼\exp\left(-\frac{KV}{k_{B}T}\left(H_{S}-H\right)/H_{S}\right)$$

where 𝐾 and 𝑉 are the anisotropy energy density and core volume, respectively, 𝐻_S_ is the characteristic field required to saturate the average nanoparticle [26]. This field, lying between the coercivity field and the anisotropy field, depends on the angle between the applied field and the easy axis of the nanoparticle as well as the inter‐nanoparticle interaction, in turn determined by the density of the nanoparticles in the cellular microenvironment. Note that because of the distribution of the nanoparticles over the membranal surface, the coercivity field, and thus the saturation field, varies in a wide range (Figure 5). This expression reflects the fact that the effective activation barrier for magnetization reversal decreases as the applied field approaches 𝐻_𝑆_. Let 𝑛 denote the surface density of MENPs anchored to the membrane and 𝑛_Thr_ denote the minimum density required to deliver enough energy to exceed the local firing threshold in a membrane patch of area 𝐴_Loc_. The local energy per nanoparticle contributing to stimulation is represented by 𝑤, which incorporates both the magnetoelectric energy transfer and the biophysical threshold of the neuron. For sub‐threshold conditions 𝑛<𝑛_Thr_ and 𝐻<𝐻_𝑆_, the probability that a neuron in that patch fires can be approximated as:

(6) $$P∼\exp\left(-\left(n_{Thr}-n\right)wA_{Loc}/k_{B}T\right)\cdot \exp\left(-\frac{KV}{k_{B}T}\left(H_{S}-H\right)/H_{S}\right)$$

This expression highlights two independent control knobs: (i) the material and structural properties of the MENPs, captured by 𝐾, 𝑉, and 𝑤, and (ii) the neuromodulation parameters, primarily the magnetic field amplitude 𝐻, with the frequency contributing through *w*. Increasing nanoparticle density 𝑛, optimizing 𝐾 and 𝑉 to maintain strong magnetoelectric coupling on the relevant timescale, or increasing 𝐻 toward 𝐻_𝑆_ all raise the firing probability.

#### 2.1.6 Magnetostrictive Core Design

The above analysis assumes each MENP core behaves as a single‐domain particle with well‐defined anisotropy. This assumption is justified for nanoscale cores, assuming the equilibrium energy accounts for the domain wall energy and the demagnetization energy, in turn depending on intrinsic material parameters such as the magnetocrystalline anisotropy energy density 𝐾, the exchange constant 𝐴, and the saturation magnetization 𝑀_𝑆_.A basic physics estimate for the characteristic size at which a nanoparticle transitions from single‐domain to multi‐domain behavior gives a threshold diameter on the order of $\delta_{th}∼\;\frac{3\pi \sqrt{KA}}{M_{S}^{2}}$ [26, 27], which, for typical cobalt ferrite parameters, falls in the tens‐of‐microns rang, well above the sub‑50‑nm sizes relevant for MENPs, at least partially determined by the generally perceived size limitation to cross the BBB [28]. In this size regime, cores can safely be treated as single‐domain under global equilibrium conditions. A more stringent constraint arises from the domain wall thickness, $\delta_{w}\;∼\;\pi \sqrt{\frac{A}{K}}$. which sets the minimum scale over which a uniform magnetization can be maintained locally. For cobalt ferrite, this yields 𝛿_𝑤_ on the order of 30 nm, implying that cores in the sub‑30‑nm range are likely to remain single‐domain even when local nonuniformities are considered.

While the above analysis is sufficient to estimate basic constraints on MENP size and anisotropy for effective stimulation, it remains a simplified approximation. It assumes uniform magnetization and does not resolve how the spin configuration varies near the core–shell interface, where magnetostriction and strain transfer are most critical. A more comprehensive and predictive description requires nanomagnetic simulations that explicitly compute the geometry‐dependent, nonuniform spin distribution and include quantum‐mechanical exchange and spin–orbit interactions between adjacent spins, especially at the interface surface. Such simulations are particularly important for optimizing MENP performance, as they enable rational tailoring of material parameters and nanoparticle geometry to specific neuromodulation targets [29, 30].

Even within this simplified framework, it is clear that MENP function can be finely tuned through intrinsic core properties such as magnetocrystalline anisotropy energy, exchange constant, and saturation magnetization, as well as size and shape. Composition control provides a powerful handle on these quantities. In the core–shell MENPs considered here, the core is typically a ferrite such as cobalt ferrite in an inverse spinel lattice. Spinel ferrites consist of a close‐packed face‐centered cubic oxygen sublattice with two types of interstitial sites: tetrahedral (A) and octahedral (B). Divalent and trivalent cations can occupy these sites in different configurations, which determine the long‐range magnetic order. In the inverse spinel configuration, divalent ions occupy B sites, while trivalent ions are distributed between A and B sites. The magnetic moments on A and B sublattices, set by the specific cations, couple primarily via superexchange through the intervening oxygen 3p orbitals, leading to antiparallel alignment of A and B moments and a net ferrimagnetic state when their magnitudes differ [31]. Because the anisotropy energy, exchange constant, and saturation magnetization all derive from this cation arrangement and associated spin–orbit coupling, they can be tuned over a wide range by appropriate cation selection and synthesis conditions [26]. It is therefore essential that the synthesis process reliably produces the desired inverse spinel ferrimagnetic phase rather than structurally similar but magnetically distinct phases (for example, antiferromagnetic hematite‐like phases). At the nanoscale, additional complexity arises from surface anisotropy effects, which can be comparable to or even dominate bulk contributions. These surface effects depend sensitively on composition, size, and shape, and may significantly influence the observed magnetoelectric response [32, 33, 34]. Experimental and theoretical studies indicate that, for ferrites, reducing the characteristic size into the sub‑10‑nm range can drive the surface toward a conducting ferromagnetic state, altering both anisotropy and magnetostriction [35, 36]. Detailed nanomagnetic simulations that include such surface contributions and quantum effects will be crucial to fully understand and exploit these size‐ and composition‐dependent phenomena in future MENP designs.

#### 2.1.7 Piezoelectric Shell Design

The most efficient piezoelectric materials generally exhibit both high electromechanical coupling coefficients, k, and large piezoelectric charge constants, *d*_xy_, characteristics most notably found in lead zirconate titanate (PZT) and related lead‐based compounds. Although these materials display outstanding piezoelectric responses, their use in biomedical or BMI applications is restricted due to the toxicity of lead to both biological systems and the environment. Consequently, biocompatible, lead‐free alternatives have been investigated, though often demonstrating a trade‐off in piezoelectric performance relative to their lead‐containing counterparts. In this contest, choosing biocompatible piezoelectrics is driven by the requirement to efficiently connect to the magnetostrictive core materials. The widely used cobalt‐ferrite‐based core offers favorable magnetic properties but is biologically incompatible [37]. Hence, complete encapsulation with a biocompatible piezoelectric shell, such as barium titanate, is required to effectively isolate the toxic core from biological environments. Other promising shell candidates include BZT and various other doped perovskite systems such as BCZT [38, 39, 40, 41]. The shelling effect can be easily determined in biocompatibility studies, with a large improvement in viability across multiple cell‐types in the shelled nanoparticles [42]. Among these, barium titanite and its derivatives remain among the most extensively studied and well‐characterized biocompatible piezoelectrics. However, as discussed below in more detail, switching to BCZT might be a preferred pathway for future roadmap development, because it can be lattice‐matched with a fully biocompatible Fe_3_O_4_ core nanostructure with a moderate magnetocrystalline anisotropy on the order of 10^5^ J/m^3^, thus also allowing to use a relatively low AC magnetic field amplitude to control neuromodulation, eventually shortening translation to wearable devices [43, 44].

A key consideration in designing core–shell MENPs is the compatibility of their crystal lattices. Achieving close lattice parameter matching between the magnetostrictive core and piezoelectric shell enhances interfacial strain transfer, which is essential for generating a strong magnetoelectric coupling. Additionally, in the barium titanite case, maintaining the tetragonal phase of the piezoelectric shell, leading to the desired ferroelectric state, is crucial to maximize the piezoelectric response, as the cubic phase exhibits significantly reduced polarization, characteristic of the paraelectric state [45, 46]. Therefore, controlling synthesis and cooling conditions–‐particularly minimizing the tetragonal‐to‐cubic transition near 120°C–‐is essential to maintain high piezoelectric performance [47, 48]. It can be noted that, for each characteristic measurement time, with the shell volume reduction, the ferroelectric state would become unstable, thus marking the transition into the superparalectric state (equivalent to the transition into the superparamagnetic state of the magnetic core). Furthermore, the geometry of the magnetic core (e.g., rectangular prism) may influence shell crystal orientation, promoting tetragonal phase stabilization [13].

Polymeric piezoelectrics such as polyvinylidene fluoride (PVDF) also present biocompatible alternatives, though their piezoelectric outputs are generally lower than those of ceramics [49]. One crucial limitation of such polymers is potential degradation in biological environments, which could expose the underlying core and compromise biocompatibility. While replacing toxic magnetic cores with biocompatible or biodegradable alternatives may alleviate this issue, it remains a challenge to match the magnetoelectric efficiency created by using a higher piezoelectric coefficient material such as barium titanate or BCZT with polymers [21].

#### 2.1.8 Prior Published Experiments Supporting Theoretical Framework: It's All About Materials

To relate the above theoretical framework to experimental performance, several material and structural parameters of the MENPs must be quantified. These include the magnetoelectric coefficient, magnetic hysteresis loop, magnetostriction, magnetic anisotropy and coercivity of the core, the “butterfly” curve, ferroelectric loop and phase state of the shell, nanoparticle size and size distribution, core–shell interfacial quality, and biocompatibility in relevant biological microenvironments. Together, these parameters determine the efficiency of the magnetostriction–strain–polarization cascade and, ultimately, the neuromodulation efficacy and safety under a given magnetic field protocol. All the mentioned properties are not independent; rather, they exhibit strong interdependencies. For example, surface chemistry can influence both magnetic aggregation and affinity to the cellular membrane, which in turn alters size distribution and effective magnetoelectric coupling. The properties have been described in detail in recent papers related to different generations of MENPs, as described below in more detail [12, 19, 50].

Of a particular importance is the observation made through in vitro studies using two consecutive generations of the CoFe_2_O_4_@BaTiO_3_‐based MENPs [12, 50]. These two generations deferred mostly in the quality of their crystallinity, with Generation 1 and Generation 2 MENPs having their magnetoelectric coefficient smaller and larger than approximately 1 V/cm/Oe, respectively. In these experiments, primary E18 rat hippocampal neurons were cultured on glass coverslips and incubated with CoFe_2_O_4_@BaTiO_3_ core–shell MENPs at concentrations on the order of 0.5–1 µg per 10^5^ neurons, providing sufficient nanoparticle coverage to achieve near‐uniform coverage of the neuronal somata and proximal neurites. Cells were loaded with Cal‐520, a calcium‐sensitive fluorescent indicator, to report neural activity at single‐cell resolution across fields of view containing hundreds to thousands of neurons. Neural activity was recorded in repeated segments consisting of baseline, stimulation, and post‐stimulation epochs, allowing within‐preparation comparison of firing patterns with and without magnetic stimulation. Magnetic stimulation was delivered using a custom coil system capable of generating bipolar AC pulses and DC offsets with field amplitudes in the 1–2 kOe range and frequencies in the 10–100 Hz band, as predicted by the M–H analysis to maximize magnetization change. In the AC condition, trains of rectangular or sinusoidal pulses (e.g., 20–50 Hz, pulse widths of 20–25 ms) were applied, with amplitudes chosen to exceed the coercive field of the MENP core, thereby ensuring that each pulse drove a substantial magnetization reversal and, consequently, a sufficiently large local electric field across the membrane. Control conditions included: (i) neurons without MENPs exposed to the same magnetic fields, and (ii) MENP‐labeled neurons without applied field, to decouple nanoparticle effects from magnetic stimulation. Neural responses were quantified using both event‐based and population‐level metrics. At the single‐neuron level, calcium transients were detected and aligned to the timing of individual magnetic pulses to assess activation rise time synchronization and latency in the sub‐25‐ms range (limited by the calcium dye response) with 1,200 Oe, 25‐ms pulses at 20 Hz [51]. At the network level, firing rates and synchrony were analyzed over baseline, stimulation, and recovery windows using statistical models that account for correlations among neurons in the same field of view. This framework allowed classification of each stimulation attempt as producing no change, a modest but significant modulation, or a large change in activity, similar to the categorization used in earlier in vitro MENP neuromodulation studies.

Though conducted under equivalent conditions, the two generations of MENPs led to significantly different results. With the first‐generation nanoparticles, only subthreshold activation was triggered. In contrast, with the second‐generation MENPs, suprathreshold activation, i.e., in the form of action potentials, was triggered. This comparison shows that Generation 1 MENPs could provide energy large enough for subthreshold activation, although not sufficient for inducting action potentials. In contrast, the improved Generation 2 MENPs could provide enough energy to induce action potentials. The comparison demonstrates the importance of the materials science perspective, suggesting that further enhancing the MENPs’ key materials properties would further improve neuromodulation efficacy and control.

#### 2.1.9 Prior Published Experiments Supporting Theoretical Framework: Matching Magnetic Fields to MENPs’ Properties

In another independent in vitro study, using the above Generation 2 MENPs and primary E18 rat hippocampal neuronal cell culture, a conducted experiment supported the theory described in the above Section 2.1.4 [52]. The experiment compared neural activation for three different AC magnetic field strength values of 1, 1.4, and 1.7 kOe, respectively, plus a control measurement at a 1.7‐kOe magnetic field with no MENPs. In perfect agreement with the above theoretical framework (e.g., Equation (6)), the experiment showed that the applied magnetic field required to efficiently stimulate neurons scaled with the saturation magnetic field of the nanoparticles. Indeed, the saturation field, exceeding the (extrinsic) coercivity field and below the (intrinsic) anisotropy field, measured for the nanoparticles at densities corresponding to the experiment, was found to be on the order of 1.5 kOe, corresponding to the average saturation field according to the M–H loop of the nanoparticles [12]. With barely firing measured at 1 kOe, a significant burst of firing neurons was noted at 1.4 kOe, further drastically increasing at 1.7 kOe, while not present in the case with no nanoparticles.

#### 2.1.10 How Frequency Can be Used To Select between Excitation and Inhibition of Local Neural Activity

**FIGURE 6:**
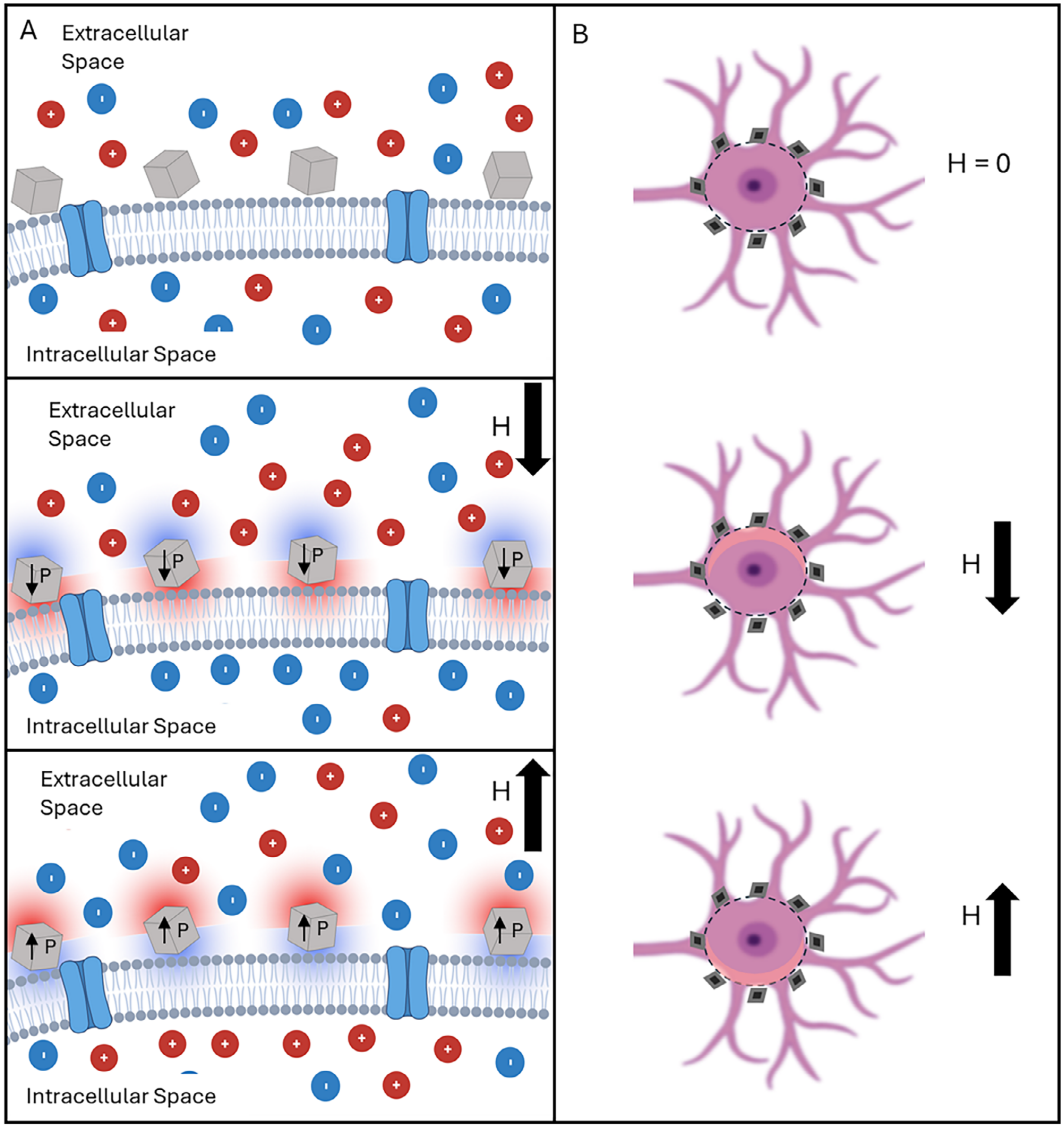
Application of AC and DC magnetic fields could break the typical spherical symmetry of the neural firing process. A) High‐level illustration showing how application of an AC magnetic field, with a frequency, f, chosen so that the effective measurement time, 1/f, is comparable to the characteristic ion channel activation time, application of the AC magnetic field effectively creates a resonance condition, in turn causing a local neural excitation: (top) no field applied, the nanoparticle remains in a relatively depolarized state, only with a slight non‐zero polarization, the membrane potential is close to its resting value; (middle) negative magnetic field orientation polarizes the nanoparticle's electric dipole, the nanoparticle's electric field further increases the membrane potential; (bottom) reversing the magnetic field orientation polarizes the nanoparticle's electric dipole moment in the opposite orientation to induce an electric field that locally depolarizes the membrane, thus effectively reducing the local membrane potential. B) Illustration to show how application of a DC magnetic field can inhibit neural activation by breaking the typical spherical symmetry of the neural firing process. The top image shows the average neuronal cell with MENPs lined up uniformly and symmetrically with respect to the spherical shape at zero field, *H* = 0. At zero field, the magnetic core of each MENP might be close to being demagnetized. As a non‐zero field is applied, the symmetry is broken along the applied field direction. Depending on the field orientation, either the top or bottom part of the cell is experiencing a significantly increased effective membrane potential (shown by red regions), thus significantly reducing the ability to form an action potential.

The capability to control the type of targeted neuromodulation by MENPs, e.g., selecting between excitation or inhibition, via the applied magnetic field spatiotemporal pattern, e.g., its frequency, is another property made available owing to the ME effect. The underlying physics is described below. To explain the frequency effect of the MENP neuromodulation, it is assumed that the nanoparticles are uniformly distributed over the neuronal membrane surface (Figure 6). In this case, as described above, each MENP effectively becomes an integral part of the membrane's ion‐gating process. Magnetic field‐controlled local electric fields from magnetoelectric nanoparticles directly modulate bidirectional ion transport across the cell membrane. Consequently, the electrochemical equilibrium underlying this ion flow becomes directly dependent on this magnetic field's spatiotemporal pattern. With this relatively straightforward physics being the essence of the MENP‐based control, the required specific spatiotemporal pattern depends on the specific biological process to be controlled. For example, if the applied magnetic field frequency is chosen to match the reciprocal of the characteristic ion channel activation/relaxation time, this frequency would force a natural, biological, resonance, promoting the specific event associated with the ion channel activation, e.g., an excitation, likely in the frequency range of 10–100 Hz, depending on the cell type and local microenvironment conditions, e.g., pH level and others [53, 54]. In contrast, if the frequency is chosen to oppose the targeted biological resonance, the event could be inhibited by this frequency. In a trivial example, applying a DC magnetic field to a spherically shaped neuron will break the spherical charge symmetry of the membrane. As a result, one half of the cell would effectively have a significantly larger membrane potential compared to the other half, leading to an energy equilibrium that cannot sustain firing. In other words, application of a DC magnetic field could be used to induce targeted inhibition of neural activity. Below, we describe in vitro studies that confirm this frequency dependence. This feature of MENPs could be particularly important to treat patients with Epilepsy.

#### 2.1.11 Prior Published Experiments Supporting Theoretical Framework: Using Magnetic Field Frequency to Toggle Between Excitation and Inhibition

A recent in vitro study with Generation MENPs 2, using primary E18 rat hippocampal neurons, confirmed the predicted frequency dependence and the ability to toggle between local excitation and inhibition [12]. AC fields at frequencies matched to typical biological channel activation times (10–100 Hz) promoted excitation by repeatedly aligning MENP‐generated fields with gate opening dynamics, as described in Section 2.1.7. In contrast, as predicted, DC fields of comparable amplitude suppressed neural activity.

In an another in vitro study, neural activation in the same neuronal cell culture was comparatively studied with CoFe_2_O_4_@BaTiO_3_ MENPs of different coercivities under equivalent field conditions [52]. Together, these in vitro experiments provided a quantitative test of the key theoretical predictions: (i) neuromodulation efficacy depends sharply on the field amplitude relative to the MENPs’ M–H loop; (ii) the field frequency and waveform can bias the response toward excitation or inhibition; and (iii) properly engineered MENPs can elicit action potential‐like signals with timing and magnitude comparable to those produced by conventional electrical stimulation in the same preparations.

### 2.2 MENP‐BCI: Recording Mode

**FIGURE 7:**
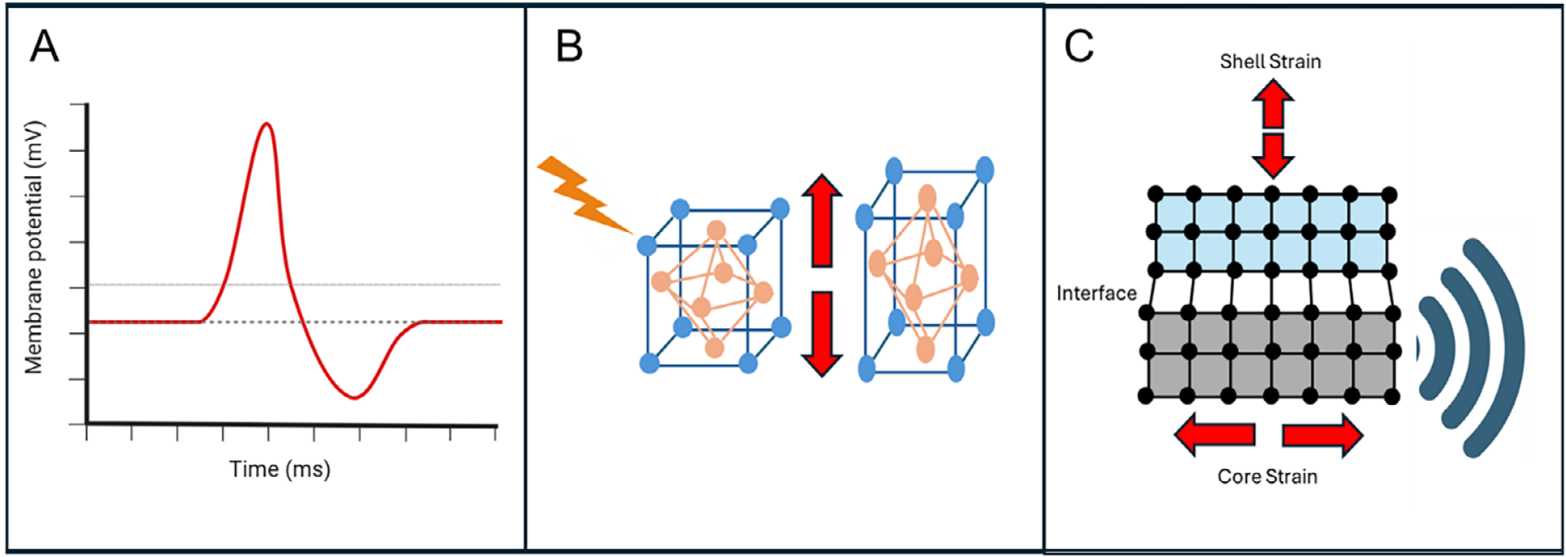
Sequence of physical processes in the core–shell MENP structure during each recording event: (A) Neural firing causes an electric field spike, leading to (B) polarization change in the shell according to the P‐E hysteresis loop; in turn, the polarization change causes a strain according to the “butterfly” curve; (C) the strain propagates through the lattice‐matched interface to the core; then, according to the inverse magnetostriction (Villari) effect, the transferred strain induces a magnetization change in the core, which in turn can be detected via magnetometers.

Whereas neuromodulation relies on the direct magnetoelectric effect (magnetic field → electric field), MENP‐based recording exploits the converse effect: local electric fields generated by neural activity modulate the polarization of the piezoelectric shell, which in turn alters the magnetization of the core and produces a detectable magnetic signal. The sequence of physical processes is therefore the reverse of the stimulation cascade: transmembrane voltage fluctuations drive strain in the piezoelectric shell, this strain is transferred across the core–shell interface, and the resulting magnetostrictive response in the core changes its net magnetization, which can be sensed by external magnetometers (Figure 7). However, it is noteworthy that, despite the role reversal, it is the magnetic field that is used to wirelessly transfer energy in both neuromodulation and recording modes. Given the characteristic time constant in this core–shell type of nanostructures is in the nanosecond scale, i.e., substantially faster than the characteristic time of neural activation mechanisms in the millisecond scale, the MENP‐BCI recording mode could enable neural recording in real time, with a spatial resolution limited by the ability to localize the applied magnetic field energy, potentially in the sub‐millimeter size range.

#### 2.2.1 Ferroelectric Hysteresis and Converse ME Coupling

The converse ME response is governed by the ferroelectric hysteresis of the piezoelectric shell rather than the magnetic hysteresis of the core. In this case, the first relevant loop is the polarization–electric‐field (P–E) dependence. During a neural activity, an electric field is induced in the vicinity of the nanoparticles anchored to the membrane. This electric field changes the shell's polarization, which then generates strain via the piezoelectric effect. This strain propagates into the magnetostrictive core and induces a small but measurable change in its magnetization because of the inverse magnetostrictive effect, a.k.a. Villari effect. In turn, this change is measured by a magnetic sensor, thus providing the magnetic readout channel. Hence, to enable predictable recording, the shell must operate in a regime in which its polarization responds both linearly and reversibly to relatively small neural field fluctuations, without being trapped in saturated ferroelectric states that would suppress sensitivity [55]. Finally, as mentioned above, like in the neuromodulation mode, it is the magnetic field that wirelessly transfers energy in the recording mode. Hence, control of the MENP's magnetic characteristics remains critical for maximizing the recording efficacy.

#### 2.2.2 Sensitivity, Stability, and Noise Constraints

Compared to the neuromodulation mode, the recording mode imposes stricter requirements on the MENP operation, because the relevant signals from endogenous neural potentials are relatively small and broadband. Typical extracellular voltage fluctuations are on the order of tens to hundreds of microvolts, and even transmembrane potentials of tens of millivolts must be transduced into magnetization changes that remain detectable after propagating from the brain to an external sensor. To resolve these signals, the converse magnetoelectric response must be both highly sensitive and reproducible over time.

In the recording mode, the superparaelectric transition of the shell is analogous to the superparamagnetic transition of the core during the neuromodulation mode, although the superparamagnetic transition remains important also for the recording mode. The energy stored in the ferroelectric hysteresis loop should be larger than the thermal noise to ensure that the local electric field due to neural activity can cause a non‐zero polarization change, large enough to lead to a non‐zero strain, during the characteristic neural activation time on the order of one millisecond. Then, this strain propagates through the core–shell interface to the core, leading to the inverse magnetostriction effect, a.k.a. the Villari effect, equivalent to applying a local magnetic field through the spin‐orbit coupling, in turn leading to the magnetization change (Figure 7). During this event cascade, on one hand, the larger the ferroelectric coercivity field the larger the polarization switching threshold, clipping relatively small neural signals and introducing nonlinear distortions. On the other hand, the smaller ferroelectric coercivity field is often reflective of the smaller piezoelectric effect, in turn reducing the overall magnetoelectric effect. Arguably, given our current electronics capabilities, it would be beneficial for the piezoelectric shell to operate in a near‐linear region of its P–E loop, where small changes in the membrane potential produce proportionate changes in polarization and strain, leading to a magnetization change that is detectable by a magnetometer, while the magnetic core exhibiting minimal spontaneous switching on the timescale of interest. Thermal fluctuations that drive superparaelectric and superparamagnetic transitions add noise and drift, degrading signal quality, especially with reducing the size of the nanoparticle. Note that, despite the opposite direction of the cascade events, the superparamagnetic limit of the core still plays an important, likely dominant, role; the spin‐orbit coupling serves as the energy barrier that determines how efficiently the magnetization (caused by the spin) will follow the strain (caused by the orbit). Hence, because of the superparamagnetic transition dependence on the frequency, the recording mode depends on the frequency, like the above neuromodulation mode (Figure 4). These considerations define the required combinations of core and shell sizes, anisotropy, shell thickness, and operating frequency more tightly than in the stimulation case, and they motivate the use of highly sensitive, low‐noise magnetometers (such as optically pumped magnetometers or SQUIDs) to recover weak MENP‐generated signals at practical stand‐off distances. However, these considerations also provide a new, flexible, platform to control neuromodulation and recording properties using the activation frequency, thus allowing more flexibility to develop new applications. For example, the emergent magnetic particle imaging (MPI) technology is a promising option for providing a powerful, preexisting tool to leverage the converse ME effect of MENPs to enable neural activity mapping in real time [16].

Ideally, if MENPs are integrated with existing magnetic imaging techniques such as magnetic resonance imaging (MRI) or the recently emerged MPI, with a temporal response on the order of 1 msec, the electric firing in the brain could be mapped in real time [56, 57, 58]. Recently, Comsol‐based finite element method calculations were conducted somewhere else to show that MPI could be used to map neural activity via MENPs [16, 59]. Another basic physics study was conducted to consider a nanoscale model that accounts for the single‐neuron microstructure, including both conducting (intra‐/extra‐cellular space) and dielectric (membrane) domains. The typical local electric field variations in the two domains are on the order of 10^5^ V/cm and 1 V/cm, respectively [60]. In the former case, due to the capacitive nature of the dielectric membrane, only a small fraction of this relatively large field across the membrane would leak out in the immediate vicinity of the membrane during each firing event. Free ions in the conducting spaces would screen out the transient electric field. This would happen relatively rapidly, with a characteristic time on the order of a millisecond, and with the characteristic screening length (the Debye length) in the sub‐1‐nm range, unless the nanoparticles are anchored to the membrane [61]. It is straightforward to evaluate the magnetic field generated by MENPs due to this spiking local electric field accompanying each firing event. The average magnetic field above the skull due to neural activity in the whole human brain is known to be on the order of 10^−9^ Oe (100 fT) [62]. This value can be used as a reference or “noise floor” to evaluate the efficacy of the MENP‐based recording. Such a field value can be detected using the conventional optical pump magnetometer (OPM)‐based MEG system [63]. Assuming the nanoparticles have their typical specifications and considering the case with MENPs anchored to the membrane, it was theoretically predicted that only approximately 10^8^ nanoparticles (∼10 ng) would be required to generate a magnetic field comparable to this noise floor value [15]. In other words, only 10^8^ MENPs can generate a magnetic field with the magnitude comparable to that generated by the whole human brain without nanoparticles. In turn, such a small number of MENPs could resolve the neural activity from a region in the brain as small as 1 mm^3^. For comparison, it can be noted that in previous in vivo studies, they have placed over 10^10^ MENPs in the whole mouse brain to wirelessly modulate neural activity, with no emphasis to ensure the nanoparticles are anchored to the membrane [14, 64, 65]. The required anchoring of the nanoparticles could be achieved through a special pre‐administration field treatment of the nanoparticles, as described in a comprehensive numerical modeling study [66]. In other words, this conservative theoretical analysis gives an encouraging prediction. Moreover, it is still not a fundamental limit of the MENP‐based recording.

**FIGURE 8:**
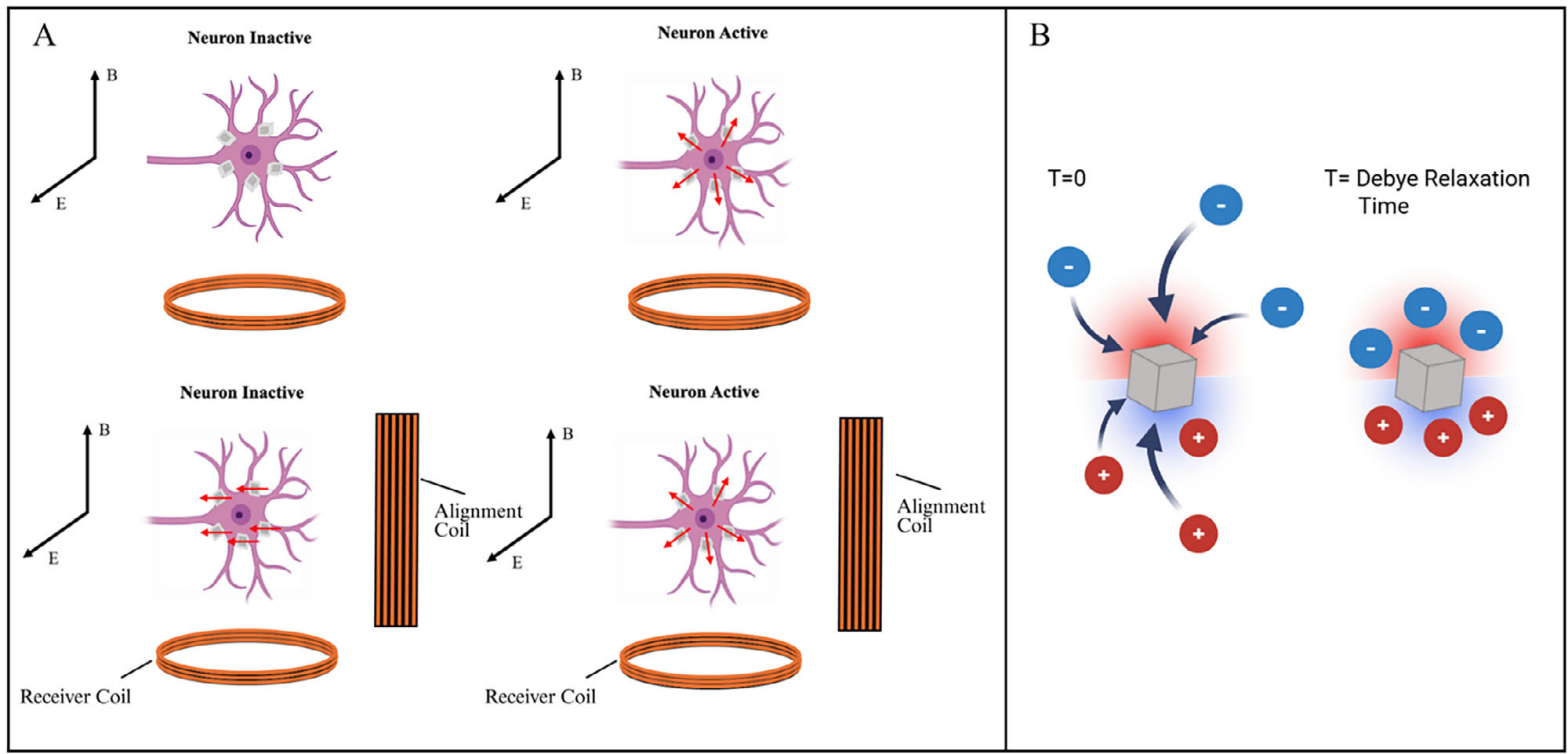
(**A**) Illustration of the active recording mode. (B) Illustration of the frequency effect on the MENP‐BCI real‐time active recording mode due to a finite (non‐zero) time it takes for the equilibrium Debye condition to set. If the frequency exceeds 1/*T*, where *T* is the time it takes to establish the Debye equilibrium, typically exceeding 1 kHz, then the MENP can detect a significant local electric field at a characteristic length limited by the average nanoparticle size.

For example, assuming the active recording mode (instead of the above passive mode) (Figure 8A), the above projection would be even more promising. In the active more, all the nanoparticles are synchronously biased by an externally applied AC magnetic field, with a relatively small strength, albeit at frequencies sufficiently large to detect the response below the characteristic biological relaxation time in the sub‐10‐msec range, known as the refractory period, e.g., at *f* > 0.1 kHz [67]. (Furthermore, to eliminate any harmful interference with biological mechanisms, the MENP activation frequency preferably must exceed the frequency spectrum of biological neural activity.) This is one of the key engineering innovations made available by the MENP BCI. Due to the converse ME effect, during each firing event, the local electric field spike due to the membrane potential reversal would be sufficient to induce a significant (detectable) magnetization change. As a result, during each firing event, the synchronous AC biasing of the nanoparticles exposed to the external magnetic field would be destroyed, leading to a significant signal‐to‐noise ratio (SNR) increase, as required for imaging of neural activity in real time.

To further boost the SNR, as required for the described active recording mode, the frequency of the activation magnetic field can be adjusted to control the penetration length of the transient electric field spiking during each neural firing event. Indeed, according to the aforementioned physics, there are two distinct time regions that occur after each firing event (Figure 8B). The first region is the transient state, lasting for a time on the order of one millisecond, which is required for establishing the Debye equilibrium. During this transient state, a substantial electric field would leak out from the membrane as far as tens of nanometers away, in other words, significantly farther than the Debye length. The exact penetration depth scales with the average nanoparticle size [19, 68]. The second region, following this transient state, is the equilibrium state, lasting for the dominant part of the action potential duration on the order of one to ten milliseconds. During this state, the electric field penetration cannot exceed the Debye length. Hence, the presence of the two temporal regions establishes a pronounced frequency dependence of the local penetration depth. If the frequency exceeds 1/*T*, where *T* is the time it takes to establish the Debye equilibrium, typically exceeding 1 kHz, then the MENP's field penetrates significantly farther away from the nanoparticle, with a characteristic penetration depth limited by the average nanoparticle size. As a result, the efficacy of the active recording mode can be substantially improved at active field frequencies exceeding 1 kHz. As for the lower end of the spectrum, like in the neuromodulation mode, the frequency is limited by the superparamagnetic limit, with the exact value depending on the nanoparticle size. Using this ab initio analysis, according to the active recording mode, using the inductive readout scheme, the signal variation through the receiver coil during each firing event could be evaluated according to this expression:

(7) $$\Delta V_{RA}=\frac{\mu_{0}N_{R}N_{T}X}{R_{R}R_{T}}\;\left(\frac{a\Delta E}{\Delta H}\right)v\omega I_{0}$$

where Δ*H* is the magnetic field variation and Δ*E* is the local electric field induced at the nanoparticle location due to the sinusoidal current *I* = *I*_0_sin(ωt) through the transmitter coil, *v* is the volume of the region under measurement, *N*_R_ and *N*_T_ are the numbers of turns and *R*_R_ and *R*_T_ are the radii of the receiver and transmitter coils, respectively, *X* is a geometric, unitless, function that depends on the geometry of the receiver and transmitter as well as the distances from the selected nanoparticle regions to the receiver and the transmitter. It can be noted that although this analysis was conducted for the inductive receiver, it could be easily modified to match any other magnetometer type, e.g., OPM, tunneling magnetoresistance (TMR) sensor, spin‐exchange relaxation‐free (SERF), nitrogen‐vacancy center (NV center) or another. Furthermore, the active recording mode of MENP recording might be suitable for being integrated into the above MPI technology. The typical operation frequency of MPI is usually in the range of 25 kHz. According to the above underlying physics, if MENPs are used instead of the typical MPI nanoparticles, such as SPIONs, the MPI signal would be modulated by the electric field due to local neural activity, thus enabling mapping of neural activity in real time, with the MPI resolution often in the sub‐1‐mm range. In this case, it would make sense to tailor the MENPs’ properties so that they remain in the superparamagnetic state during the navigation stage, while being switched out from the superparamagnetic state, and thus having a non‐zero ME effect, during the imaging step, via frequency selection.

## 3 Discussion

The development of a wireless, high‐resolution, bidirectional BCI has long been a goal in neurotechnology. Core–shell MENPs can bridge this gap by efficiently converting magnetic fields into local electric fields (and vice versa) at the nanoscale. By rigorously accounting for the nonlinear physics of the magnetostrictive and piezoelectric components, we show that MENPs can achieve targeted neuromodulation with signal response functions comparable to those by the conventional electrode‐based stimulation. The fact that the neuromodulation can be controlled just by magnetic fields, without using any bio‐reagents and consequently leading to unwanted side effects, is a significant asset in the future development of BCI. With MENPs, the neuromodulation is controlled by the ability to localize magnetic field energy, with the strength and frequency of the field chosen based on properties of the nanoparticles, e.g., the magnetocrystalline and/or shape anisotropy, coercive magnetic and ferroelectric fields, magnetic and ferroelectric resonances. In turn, such materials properties can be controllably varied in a relatively broad range to ensure magnetic fields with spatiotemporal profiles of “easily” achievable strengths and frequencies could be used to enable desired neuromodulation outcomes. To date, the spatial and temporal resolutions under investigation have been on the order of sub‐millimeter and sub‐millisecond scales, respectively. Arguably, these specifications might be sufficient to enable wireless BCI with a record spatial resolution in real time, particularly if integrated with MPI. Moreover, it is noteworthy, these specifications remain far from fundamental limits to control magnetic fields, thus further substantial advances must be expected. Further detailed studies, likely with further advancement of materials, help of machine learning tools, and using metastable physics to control local magnetic field energy would need to be conducted to understand specifics of required spatiotemporal field profiles.

The MENP approach offers distinct advantages over current wireless neuromodulation technologies. Unlike optogenetics, which remains the gold standard for cell‐type‐specific control in research, MENPs do not require genetic modification of the target tissue, removing a major barrier to clinical translation [9, 69]. While optogenetics relies on light delivery via implanted fibers or µLEDs (with limited penetration depth), MENPs are activated by magnetic fields that pass transparently through the skull and brain tissue, enabling non‐invasive deep‐brain access. Recent in vitro studies indicate that MENPs can drive calcium transients and action potentials with efficacy comparable to optogenetic actuators, but with the added potential for bidirectional operation with the same nanoparticle platform. However, it must be noted that it had taken us and our colleagues across the globe more than a decade after the conception of the idea in 2011, to turn it into a viable technology.

The reason for the relatively long development is the engineering complexity due to the non‐linear physics of the underlying mechanisms. Treating MENPs as simple linear transducers fails to capture their behavior in biological systems. The stimulation efficacy depends critically on the interplay between the applied field amplitude, frequency, and the nanoparticle's coercive and anisotropy fields and other parameters. Our theoretical analysis and experimental results from multiple independent labs confirm that these nonlinearities are not merely nuisance parameters but formidable control knobs. For instance, the superparamagnetic transition sets a fundamental frequency limit for hysteresis‐dependent magnetostriction and, consequently, the ME effect, while the ability to toggle between excitation and inhibition via the field frequency selection exploits the specific symmetry‐breaking properties of the MENP–membrane interaction. To fully seize the potential of this approach, these non‐linearities need to be considered not only by maximizing the ME coefficient but also by ensuring that the applied magnetic field's strength and frequency are tailored to both nanoparticles’ properties and biological media characteristics.

Based on the presented analysis, we could conclude that the most important future development in the area of MENP‐BCI will likely stem from advanced materials development. For example, in agreement with the presented theory and supporting in vitro measurements, improving the quality of the nanoparticles alone was sufficient to go from sub‐threshold [50] to supra‐threshold neural activation, leading to induction of action potentials [12]. As predicted by the presented theory, the in vitro experiments prove that to achieve high‐efficacy neuromodulation, the applied magnetic field strength needs to be scaled with nanoparticles’ properties such as the coercivity and magnetocrystalline anisotropy fields – extrinsic and intrinsic parameters, respectively [52]. Furthermore, because the coercivity field depends on the applied magnetic field frequency, the frequency can be used to control the neuromodulation process. The latter is a very significant merit of MENPs. It allows to use relatively small nanoparticles, e.g., in the 10‐20‐nm size range, with the core in the 5–15‐nm range. Under typical low‐frequency conditions (below approximately 10 Hz), core materials such as iron oxide, would be in the superparamagnetic state. In turn, being superparamagnetic, they will not tend to agglomerate and thus, with the size being comparable to the membrane thickness, would be more likely to target the membrane to minimize the electrostatic energy [66]. Only then, after the nanoparticles target the membrane, a higher frequency AC magnetic field component can be applied, e.g., in the form of low‐frequency trains of bipolar pulses with a relatively fast ramp time (∼< 1 msec, equivalent to a frequency component with *f* > ∼1 kHz) can be applied to turn on the ME effect, thus causing the desired neuromodulation.

Recently, significant progress has been achieved along this path to develop novel core–shell MENP materials [70]. For example, using lower‐anisotropy and thus, lower‐magnetostriction core materials, at the cost of using piezoelectric materials with a higher piezoelectric coefficient, could allow to use a substantially lower magnetic‐field‐strength, e.g., in the sub‐1‐kOe or even sub‐0.1‐kOe range. In turn, using a lower magnetic field strength for neuromodulation would make implementation of the approach substantially less complicated and energy‐efficient, thus accessible even in small clinics and eventually translatable into a small form factor device suitable for wearable applications.

Conversely, using lower‐anisotropy materials will be important for the recording mode. Particularly, using the above frequency control of the superparamagnetic state, and consequently the ME effect, provides a unique opportunity to integrate MENPs into the MPI platform. In turn, leveraging the combined properties of MENPs and MPI will enable high‐resolution neural activity map of the entire brain in real time.

Another important milestone would be to implement adequate electromagnetic systems to control both targeted MENP neuromodulation and recording modes. For example, the magnetic field frequency could be used to toggle between neuromodulation and recording modes, especially using the active recording mode, while the amplitude being used as an “On/Off” switch for both modes, with each mode having its own amplitude range [19]. Furthermore, selecting different frequencies can also be used to separate targeted excitation and inhibition events during the neuromodulation mode, as proven through presented in vitro measurements. In turn, not requiring molecular biomarkers to target specific cell types, often leading to undesired side effects, would further substantially extend the impact of the MENP‐BCI approach.

As for the temporal response of the core–shell nanoparticles, it is fundamentally limited by intrinsic physical resonances of both core and shell, as well as the core–shell interface, including the ferromagnetic, dielectric and mechanical resonances. Coincidentally, all these resonances for the size range of MENPs under study, take place in gigahertz ranges, equivalent to the sub‐nsec time scales, thus significantly exceeding the temporal response required for neural activity imaging in real time, i.e., in the sub‐msec range [71, 72, 73]. This is particularly relevant for the recording mode, currently discussed only theoretically. In this case, an externally applied magnetic field frequency, in a range different from the typical neural activity range, e.g., significantly exceeding 1 kHz, could be chosen to produce an active recording approach, optionally integrated with the MPI platform, during which the frequency harmonics of neural activity modulate the activation frequency.

## 4 Future Projections

The theoretical models, with experimental validations discussed for the neuromodulation mode, demonstrate that core–shell MENPs provide a scalable, wireless two‐way BCI platform capable of targeted neuromodulation and recording. However, translating this physics‐based proof‐of‐concept into a clinical reality requires navigating a complex space defined by nonlinear magnetoelectric coupling, biological constraints, and system‐level engineering, albeit, with no fundamental limitations in the near future. Based on our analysis, we identify three critical vectors for future development.

### 4.1 Advanced Materials Engineering for High‐Efficiency Coupling

The most significant performance gains will likely come from optimizing intrinsic properties of MENPs. As our theoretical framework predicts, maximizing the magnetoelectric coefficient directly lowers the magnetic field threshold for neural activation. While current CoFe_2_O_4_@BaTiO_3_ nanoparticles are effective, next‐generation designs should exploit materials with higher piezoelectric coefficients, such as BCZT, which offers piezoelectric responses higher than that in barium titanite, thus allowing to use core materials controlled by lower fields, e.g., lower anisotropy ferrites, preferably not containing toxic elements like cobalt [74].

Coupling such high‐performance shells with cores made of lower anisotropy materials, compared to cobalt ferrite, e.g., manganese or nickel ferrite or just iron oxides, would enable robust magnetostriction at much lower field strengths, drastically simplifying the requirements for external electromagnetic coils and reducing power consumption. Furthermore, eliminating cobalt from the core composition mitigates toxicity risks associated with potential leaching, addressing a key safety concern. In addition, lowering the anisotropy would allow to use MENPs in the superparamagnetic state, thus minimizing risks of agglomeration during navigation and targeting, while bringing the nanoparticles out of the superparamagnetic state only during activation, for neuromodulation or recording, by applying magnetic fields with adequate frequency components. Future synthesis efforts must also focus on surface engineering. Functionalizing MENPs to enhance hydrophobicity and membrane affinity, i.e., “anchoring” the nanoparticles to the membrane, would maximize the coupling of local electric fields to ion channels, ensuring that every actuation event translates efficiently into membrane depolarization.

### 4.2 Precision Electromagnetic Control

Beyond materials, the external control system offers a powerful, underutilized degree of freedom. Published in vitro studies confirm that the frequency and waveform of the applied magnetic field can selectively drive excitation or inhibition without changing the nanoparticle composition. Future BCI systems should leverage this by implementing adaptive pulse sequences, for example, using resonant AC bursts to activate specific neural populations and DC offsets or detuned frequencies to suppress pathological activity (e.g., in epilepsy). Furthermore, theoretical models show that using the active recording mode with the external activation magnetic field at a frequency above 10 kHz could significantly increase the SNR to the level required for real‐time neural recording with a spatial resolution better than 1 mm^3^, especially when integrated with the MPI platform. Developing compact, wearable coil arrays capable of generating these complex spatiotemporal field patterns will be a key engineering milestone.

### 4.3 Reciprocity Limitations and Recording Challenges

While neuromodulation is relatively mature (supported through in vitro and in vivo measurements [12, 13, 14, 64]), MENP‐based recording faces distinct physical challenges, albeit achievable, according to our theoretical models. The reciprocity between neuromodulation and recording is imperfect. Where neuromodulation is limited by the magnetic core's superparamagnetic transition, recording is constrained also by the piezoelectric shell's ferroelectric stability and “superparaelectric” limit. Nevertheless, arguably, the superparamagnetic limit plays an important role for both modes because it is the magnetic field which is used for wireless energy transfer with this BCI type in both directions. In turn, the characteristic time of the superparamagnetic transition, depending on the nanoparticle's core size and the spin–orbit coupling energy, defines the lower frequency limit required for MENP‐BCI to operate. As for the higher frequency bound, our analysis suggests that the intrinsic temporal response of MENPs extends into the gigahertz regime (governed by ferromagnetic and dielectric resonances), far exceeding the millisecond‐scale requirements of neural signaling [71, 72, 73]. The bottleneck for recording is therefore not bandwidth but sensitivity and signal‐to‐noise ratio. Realizing the full potential of bidirectional interfacing will require sensitive magnetometer arrays (e.g., OPMs) and advanced signal processing to recover MENP signatures from biological background noise. Basic physics analysis shows that using an active recording mode can significantly improve the recording efficacy, ideally showing that with MENPs, SNR values comparable to those achieved today from the whole brain using conventional OPM‐based MEG systems could be achieved from a region as small as 1 mm^3^. Furthermore, MENPs, integrated with the emerging technology of MPI, will likely enable real‐time recording of neural activity with a sub‐1‐mm^3^ spatial resolution, requiring only less than 1 µg of biocompatible, and in the future, optionally, biodegradable, nanoparticles.

## 5 Conclusions

By rigorous analysis of the nonlinear physics governing MENP operation, we have established a theoretical framework for rational design of wireless bidirectional neural interfaces. We demonstrated that properly engineered MENPs can deliver millisecond‐precision neuromodulation in deep‐brain and cortical targets, bypassing the invasiveness of electrodes and the genetic burden of optogenetics. With a clear roadmap for materials optimization and system integration, MENP technology is poised to evolve from a laboratory tool into a clinically viable platform for treating neurological disorders and decoding brain function.

## Funding

Defense Advanced Research Projects Agency (DARPA) under contract number N66001‐19‐C‐4019, National Science Foundation (NSF) under grant number ECCS‐211082, and the Sylvester Comprehensive Cancer Center (SCCC) intramural funding program under the core National Institutes of Health (NIH) grant 5P30240139‐02.

## Conflicts of Interest

In 2015, Sakhrat Khizroev and Ping Liang co‐founded a tech start‐up Cellular Nanomed, with the purpose to create a first wireless MENP BCI.

## Data Availability

The data that support the findings of this study are available from the corresponding author upon reasonable request.
